# Improved quality of life after Ibandronic acid infusion in patients suffering from diffuse sclerosing osteomyelitis of the jaw

**DOI:** 10.4317/medoral.26761

**Published:** 2024-10-13

**Authors:** Thomas Frank, Ina Dewenter, Sven Otto, Birte Julia Siegmund, Wenko Smolka, Tim Hildebrandt, Katharina Theresa Obermeier

**Affiliations:** 1 Department of Oral and Maxillofacial Surgery and Facial Plastic Surgery, Ludwig-Maximilians-University (LMU), Munich, Germany

## Abstract

**Background:**

Quality of life research with respect to patient reported outcomes (PROs) other than pain has not yet been conducted in the field of Diffuse Sclerosing Osteomyelitis. This cross-sectional study aims to investigate changes in quality of life regarding 34 subjective parameters in patients with diffuse sclerosing osteomyelitis after intravenous Ibandronic acid administration (6mg).

**Material and Methods:**

15 patients (11 female, 4 male) with diffuse sclerosing osteomyelitis (DSO) treated with 6mg of Ibandronic acid completed the standardized questionnaires (EORTC QLQ-C30, EORTC QLQ-H&N35, OHIP-G 14) considering quality of life before and two weeks after infusion.

**Results:**

All 15 patients reported a significantly improved quality of life after administration of Ibandronate. Patients reported improvements in oral health associated quality of life as well as reduction of pain and intake of analgesics. In addition patients reported a significant improvement in fatigue, sexuality, social interactions, emotional, cognitive and role functioning. Furthermore patients reported an improvement in mouth opening, weight loss and loss of appetite as well as a reduction of speech and swallowing problems. Moreover, insomnia occurred less frequently after bisphosphonate infusions.

**Conclusions:**

The study evaluates patients subjectively benefit from a standardized Ibandronic acid regimen. A significantly improved quality of life after administration of Ibandronate was observed in all 15 patients.

** Key words:**Patient-centered outcomes research, osteomyelitis, bisphosphonates.

## Introduction

The rare disease of Diffuse Sclerosing Osteomyelitis (DSO) of the jaw has been subject to research for more than 40 years (1). DSO is described as an inflammatory bony lesion without evident clinical effect of pathogenic bacteria (2). It is seen as a form of pimary chronic osteomyelitis appearing in the jaw (3). Therefore this rather rare disease might be of rheumatic descent (4). However, pathogenesis is still unclear. In most cases the mandibula is affected (2) and there is a typical circle of acute episodes and remission of symptoms. Typically, described symptoms are trismus, pain and recurring swelling (1,5,6). Asymmetry of the face due to bone remodeling processes and consecutive permanent swelling is reported. In contrast to bacterial Osteomyelitis, there is an absence of pus or fistulae/sequestration (2).

Diagnosis of DSO is a challenge as it is a very rare disease. Leading clinical diagnose tools are the panoramic radiograph and magnetic resonance imaging (7,8). Alternating sclerotic and radiolucent areas as well as a blurred nerve canal can be found. A noticeable anamnesis can be validated by bone szintigraphy, showing an increased uptake of Technetium99m (Tc99m) (2). Quality of Life analysis making use of PROMs (Patient Reported Outcome Measures) is a valid method carried out by the patients completing standardized questionnaires, such as designed by the EORTC (European Organisation For Research And Treatment Of Cancer) (9). There are several forms that can be shortened or combined to best fit the categories a researcher wants to measure (10).

The multi-factoriality of well-being can be addressed very well with these questionnaires when they are answered from the subjective patients’ point of view, considering the fact that not all desirable results from the clinician’s perspective may seem equally important to the patients (11). Any form of a patient stating his or her health concerning a certain treatment or underlying condition without interpretation of the clinician is therefore regarded as PRO (Patient Reported Outcome) (12). In this study, the effects of a distinct therapeutic regimen for DSO patients were investigated with this method. Ibandronic acid, a high-potency sodium-containing bisphosphonate (13), was administered with a dose of 6mg intravenously when the patients were in an acute relapse of the disease with pain. This approach has already shown favorable results in a study for pain relief in DSO patients with Ibandronic acid conducted by Otto *et al*. in 2017. This drug has a rather long half-life in the bones of about 10 years (5,14). Based on previous findings, it was shown that the reduction of pain reached a maximum after two weeks. However, the aforementioned study by Otto *et al*. did only report pain before and after treatment. QoL questionnaires were not part of the study in 2017, whilst in this present study it was essential to report numerous patient-reported outcomes that affected patients’ everyday life with PROMS in order to show that Ibandronate administration following a certain protocol can lead to largely improved quality of life for DSO patients in many more ways than just pain relief. Even though there are various positive effects of bisphosphonates, in literature there is an ongoing discussion about the side effects of repeated intravenous antiresorptive drug administration, because these agents are infamous for the side effect of medication related osteonecrosis of the jaw (MRONJ) (15). MRONJ is thought to be an effect of an extremely high amount of antiresorptives in inflamed areas of the bone, which can therefore lead to bone sequestration due to compromised bone remodeling ability. Still, by now, there are no MRONJ lesions reported in DSO patients treated with antiresorptive medication. This might be explainable due to missing trigger factors such as extraction sockets with sharp bone fragments or other inflammation signs (15). To date there are no existing patient reported outcomes from DSO patients in literature, so the aim of this study is to set a baseline for future quality of life research in the field of diffuse sclerosing osteomyelitis of the jaw. By doing so, positive effects of Ibandronic acid infusion are discussed to validate the specific therapy regimen in patients suffering from DSO.

## Material and Methods

Patients who were treated with 6mg of Ibandronic acid during an acute episode of DSO of the jaw between 2021 and 2023 were included in this study. This prospective cohort study was approved by the institutional review board of the University Hospital of Munich, Germany (Munich, Germany; UE Nr 20-1096). In this investigation the guidelines of the helsinki declaration were followed.

The first inclusion criteria was primary diagnosis of DSO of the jaw, which had to be evaluated based on clinical, radiographical and histopathological findings. Only patients with legal age were implemented in this study. All patients included were treated with Ibandronic acid 6mg i.v. and had to answer the questionnaires described in the following section both pre- and post-therapeutically in order to be included in the study.

Exclusion criteria were previous history of partial or complete mandibulary resections as well as modeling osteotomies in the head and neck area caused by any other disease than DSO except displaced and/or impacted teeth. Patients with a history of chemo- or immunotherapy or radiation in any form were excluded. Patients with permanent immunosuppression were also excluded in this study.

All questionnaires were collected in an acute episode before administration of intravenous Ibandronic acid 6mg single-dose. Two weeks after intravenous bisphosphonate administration, the questionnaires were collected again.

The following QoL questionnaires were used for patients: The European Organization for Research of Cancer’s Core Quality of Life Questionnaire QLQ-C30, the same organization’s questionnaire primarily designed for head and neck cancer H&N35 and the Oral Health Impact Profile OHIP-G 14 each for a situation of an acute episode of DSO and again for the situation two weeks after therapy. The QLQ-C30 questionnaire consists of 30 questions, the H&N35 and the OHIP-G14 of 35 respectively 14 questions. Raw scores were acquired for each scale, which could consist of one or more items so raw score translates to mean of component items. The final scores were then calculated by applying a distinct mathematic formula from the scoring manuals to bring the range of each scale from 0 to 100. The questions amongst other scales concern overall quality of life, oral health associated quality of life, physical function, role function, emotional function, cognitive function, social contact, sexuality and fatigue.

Statistical analysis: No sample size calculation was needed due to the rare nature of DSO. Analysis of the collected data was conducted with SPSS® 29.0.2.0 (20) (SPSS Inc., Chicago, IL, USA). All scores were tested for normal distribution by Kolmogorov-Smirnov test and were found not to be normally distributed for the questionnaire scores of QLQ-C30 and QLQ-H&N35. For OHIP-G14, the results were normally distributed. The difference in the mean scores of each scale pre and two weeks post infusion with 6mg of Ibandronic acid in QLQ-C30 and QLQ-H&N35 were then tested with Wilcoxon test. Because of normally distributed scores in OHIP-G14, T-test for paired samples pre and post infusion was carried out for this questionnaire.

## Results

- Demographics

Overall 15 patients were included in this study. 11 of them were female (73%) and 4 were male (27%). Age at primary diagnosis was 41 +- 25 years, age when completing the questionnaires was 46.5 ± 24.5 years with an average of 43.8 years. The number of received infusions ranged from 1 to 10. None of the patients reported general or rheumatic diseases. No allergies were reported. Regular nicotine abuse could be found in 3 patients, alcohol abuse was not found in any patient. The raw scores acquired are shown in Table 1.

- Results of QoL assessment

**Figure 1:**
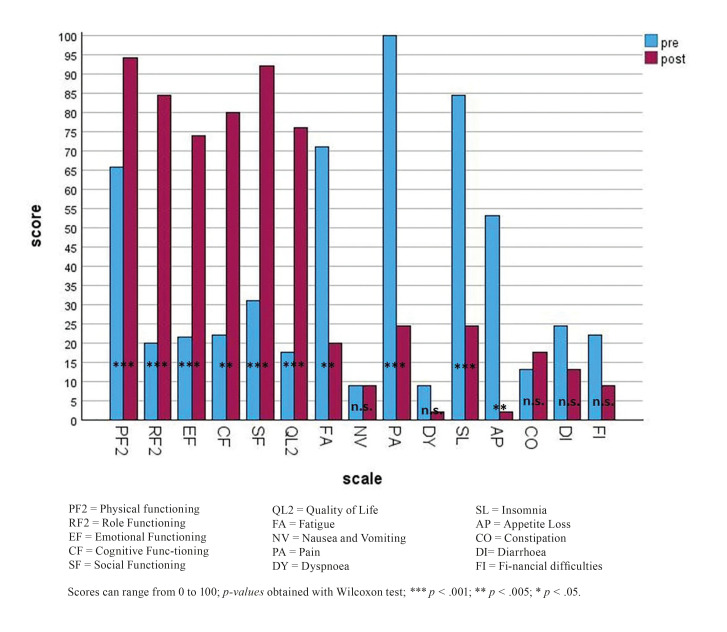
Mean scores for QLQ-C30 pre and post infusion.

Three-fold increase in patient reported quality of life within the meaning of a mean score difference of 58.33 indicated effectiveness of Ibandronic acid with a high level of significance (*p* < .001). Nearly similar results were found in terms of oral health related quality of life, which increased as indicated by an OHIP-G14 score of 25.93 less on average. (*p* < .001, Table 2). In terms of results of QLQ-C30 (Fig. 1, Table 2), social functioning improved from 31.11 to 92.22 (*p* < .001). An improvement of emotional functions by 52.22 (*p* < .001) and of cognitive functions by 57.78 (*p* < .005) was reported, also a change for the better regarding physical functioning by 28.45 (*p* < .001). Role functioning was increased three-fold to a score of 84.44 (*p* < .001). In summary nearly all of the patients were able to improve participation in social interactions two weeks after administration of Ibandronic acid. They also felt less tired (fatigue score plus 51.11, *p* < .005), slept better (insomnia minus 60, *p* < .001), and reported more appetite by a mean score of 51.11 (*p* < .005).

**Figure 2:**
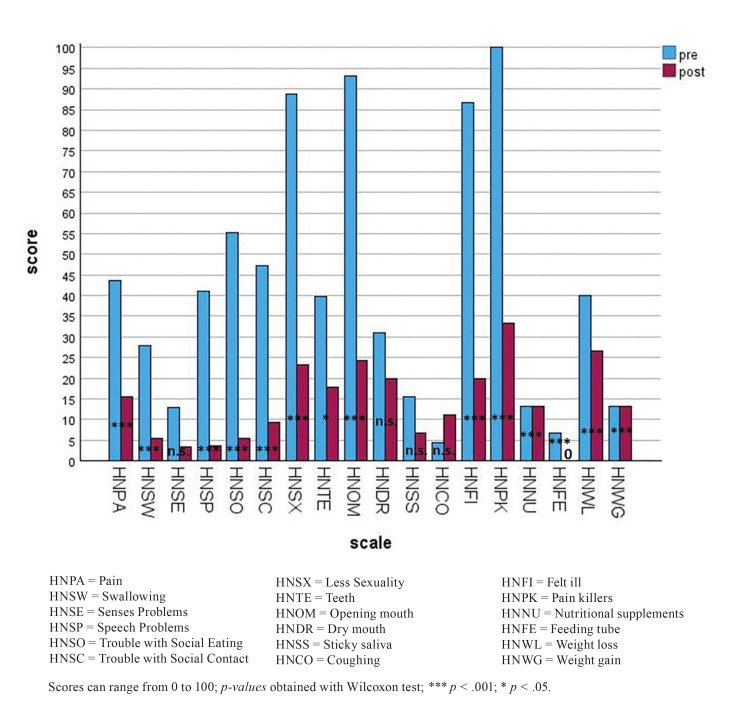
Mean scores of QLQ-H&N35 pre and post infusion.

Concerning the QLQ-H&N35 (Fig. 2, Table 2), a majority of the patients reported shame when eating with other people due to the fact of a restricted mouth opening and swelling, which also correlated with personal acceptance. Ibandronic acid infusions improved swallowing problems from 28.33 to 5.66 (*p* < .05), problems with social eating were reduced nine-fold to a score of 5.56 (*p* < .001). Mouth opening restriction was reduced from 93.33 to 24.44 (*p* < .001) with many of the patients experiencing no mouth opening restriction at all one week after Ibandronic acid infusion. Another important outcome for patients was an improvement in the ability to speak by a mean of 37.78 (*p* < .001). Social contacts and sexuality could be enjoyed more by 38.23 respectively 65.56 (*p* < .001 each). The Pain Scales of QLQ-C30 and H&N35 both highlighted positive effects of Ibandronic acid treatment in the meaning of a decrease in pain approximately four-fold respectively three-fold (*p* < .001 each). Moreover, the mode for this scale in both questionnaires was 0, which means the largest group of patients had no pain at all after treatment. Consecutively, the painkiller intake dropped three-fold (*p* < .005), leading to a median and mode of 0. Problems with teeth per se diminished by a mean of 22.22 (*p* < .05). Moreover, patients felt less ill by a factor of 3,4 (*p* < .05). On the contrary, it has to be mentioned that there were some scales descriptively showing increased scores after intravenous Ibandronic acid medication. One of them was constipation (increase of 43%). The other one was coughing (plus 150%). Increased weight loss, however, cannot be seen solely as an adverse effect of the therapy, whereas constipation appeared only moderately more often.

## Discussion

The aim of this study was to demonstrate improved quality of life in patients treated with intravenous infusion of Ibandronate 6mg during an acute episode of DSO in terms of social life, pain, symptoms and in general. Expectations of Ibandronic acid infusions as a valid tool for improvement of quality of life could be reconfirmed with this study. When it comes to patient reported outcomes, Ibandronic acid therapy showed positive effects on a variety of the DSO patients’ clinical manifestations, such as the aforementioned pain, symptoms like swallowing problems or mouth opening restrictions, which have an impact on HRQOL, as well as an improvement in social an emotional functioning.

Due to the unknown exact etiology of DSO, there have been different approaches in therapy such as hyperbaric oxygen, physiotherapy, splint therapy, surgical resection or decortication, steroids, calcitonine, antibiotics and antiresorptive drugs (5,16-18). Amongst these, antiresorptive treatment has shown the most favorable effects (6,19,20), such as symptom control, yet without existing evidence for a standardized regimen (21). In addition it has to be mentioned, that the antiresorptive mechanism leads to an inhibition of osteoclast activity but cannot be seen as a causal therapy for the underlying and to date not fully understood pathophysiologic process of DSO.

Bisphosphonates can be administered orally or intravenously in different dosages. They can be subdivided in two groups: nitrogen-containing (Zoledronate, Pamidronate, Olpadronate, Alendronate, Ibandronate, Risedronate) and non-nitrogen-containing (Disodium Clodronate) bisphosphonates (6). As an intravenous dose of 6 mg Ibandronic acid (low dose) leads to a reduction of osteoclast activity without an enhancement of osteoblast activity, it can be assumed that the osteoblast activity can be neglected (5). To quantify the clinical outcomes, PROMs, a rather new method of generating comparable results, were used (22). They are widely applied in medicine, especially in oncology (23,24) and less frequently used in dental medicine, where they are mainly common with prosthodontic research, orthodontics and geriatric dental care (25).

There is a huge variety of existing questionnaires for quality of life determination, which is why in this study the best fitting ones were chosen. OHIP-G14 was used as oral health associated quality of life indicator because it is known to be a valid tool, which is frequently used in the dental health complex and especially in oral surgery (11,25). The QLQ-H&N35 questionnaire was primarily designed for head and neck cancer patients, but it consists of a variety of symptom scales applying ideally to common DSO symptoms, such as pain, swallowing, eating problems, mouth opening and many more, which is why it was chosen for this study. The third questionnaire used was QLQ-C30, which basically consists of similar symptom scales to the H&N35, but is designed as non-disease specific and therefore assesses more generic data from the DSO patients. Moreover it was a helpful feature to compare results with H&N35. The results of this study are remarkable with respect to previously published data concerning the clinical effect of antiresorptive drug treatment, which all show positive outcomes but did not quantify specific changes in symptoms (except pain) in certain percentages (5,20). Therefore delivering exact numbers for the examined group helps in defining a baseline for future investigations. Applying potent nitrogen containing bisphosphonates, such as used in this study, could also be compared to different therapeutic regimens by investigating patient reported outcomes in order to find out the best match for improving the patients’ quality of life or specific symptoms. However, one has to be critical with interpreting subjective outcomes, because some form of placebo effect cannot be ruled out. Still, it was already shown by Van de Meent *et al*. that the reduction in symptoms in DSO patients treated with bisphosphonates shows differences among treatment and placebo group (20). Anyway the goal is to achieve what is most important to the patient, namely a stronger feeling of healthiness, which itself can lead to positive reactions in the body (this model is also referred to as patient-centered care) (26). Nonetheless, the questionnaires used for this study have some drawbacks. Cronbach’s Alpha for H&N35 is known to be reasonably high with a reliability of most scales above 70%, but this test reaches only low reliability values for Speech and Senses (27). These two scales were not of main interest in this study, so that the weaknesses of H&N35 were taken into account and the questionnaire was nonetheless used. In dentistry, Questionnaires such as the OHIP-G14 are used to examine health condition, experience with health care and routines concerning their health. All dental PROs can be categorized in oral function, orofacial pain, orofacial appearance and psychosocial impact, which was the reason why the three best fitting questionnaires from the clinician’s view had to be combined in this study in order to give the patients the chance to state their mind on most of the main quality of life altering factors influenced by DSO (28).

## Conclusions

This study provides important information on quality of life among patients with DSO, although it has some limitations. This was a single-center study and a relatively small number of patients were recruited at one center. However, to date, this is the first study assessing quality of life in DSO patients treated with Ibandronic acid. Further investigations have to be conducted to confirm, precise or refute the results of this study and to potentially find out about long term effects of antiresorptive medication between infusions and recurrence of symptoms such as pain from a patient’s point of view.

## Figures and Tables

**Table 1:** Overview of all scores for OHIP-G14, QLQ-C30 and QLQ-H&N35 (N=15).

Mean scores pre and post infusion																	
OHIP-G14	-	patient nr.	1	2	3	4	5	6	7	8	9	10	11	12	13	14	15
		score pre	35	24	33	52	22	33	35	11	44	43	27	42	22	45	22
		score post	13	1	2	8	22	8	2	0	4	2	3	2	7	14	13
QLQ-C30	PF2	pre	67	87	87	27	93	73	93	80	60	40	80	53	73	20	53
		post	100	100	100	100	93	87	100	100	100	100	93	93	100	93	53
	RF2	pre	0	33	17	0	67	0	0	67	0	0	33	0	17	33	33
		post	100	100	100	100	67	100	100	100	100	100	83	67	67	83	0
	EF	pre	25	42	17	0	50	0	0	50	0	0	67	8	25	0	42
		post	42	83	100	25	50	58	92	100	92	83	100	92	75	58	58
	CF	pre	0	33	0	0	33	0	0	67	0	0	33	0	0	100	67
		post	33	100	100	33	33	100	100	100	100	100	100	100	67	67	67
	SF	pre	17	50	33	0	67	0	33	67	50	0	33	17	67	0	33
		post	83	100	100	67	67	100	100	100	100	100	100	100	100	100	67
	QL2	pre	25	42	8	0	42	0	0	33	17	0	42	17	17	8	17
		post	83	58	83	75	42	83	75	100	83	100	83	100	75	67	33
	FA	pre	0	67	67	100	67	67	100	67	100	0	67	100	100	100	67
		post	0	0	0	67	67	33	0	0	0	0	33	0	33	0	67
	NV	pre	0	0	0	17	0	0	17	0	17	50	17	17	0	0	0
		post	0	0	0	17	0	0	0	0	17	0	0	0	0	67	17
	PA	pre	100	100	100	100	100	100	100	100	100	100	100	100	100	100	100
		post	33	0	0	100	100	0	33	0	0	0	0	33	33	33	0
	DY	pre	67	0	33	0	0	0	0	0	0	0	0	33	0	0	0
		post	0	0	0	0	0	0	0	0	0	0	0	0	0	0	33
	SL	pre	100	67	100	100	33	100	100	67	100	100	100	100	100	100	0
		post	0	0	0	67	33	67	0	0	0	0	67	0	33	67	33
	AP	pre	67	100	33	67	0	67	67	0	100	100	33	33	67	67	0
		post	0	0	0	0	0	0	0	0	0	0	0	0	0	0	33
	CO	pre	33	0	0	67	0	67	33	0	0	0	0	0	0	0	0
		post	33	0	0	100	0	67	0	0	0	0	0	0	0	67	67
	DI	pre	0	0	0	67	33	0	100	0	0	0	0	67	0	67	33
		post	0	0	0	100	33	0	67	0	0	0	0	0	0	0	0
	FI	pre	0	0	0	0	33	0	33	67	0	0	0	100	0	0	100
		post	0	0	0	0	33	0	0	0	0	0	0	0	0	0	100
QLQ-H&N35	HNPA	pre	33	25	50	58	75	50	50	25	58	42	25	42	25	58	42
		post	0	8	0	0	75	8	8	0	33	0	8	17	25	25	25
	HNSW	pre	42	42	17	67	75	25	0	0	58	17	42	17	0	25	0
		post	0	0	0	0	75	0	0	0	0	0	0	0	0	8	0
	HNSE	pre	33	0	0	33	17	0	17	0	0	0	33	33	17	17	0
		post	0	0	0	0	17	0	0	0	17	0	0	0	0	17	0
	HNSP	pre	44	44	44	67	11	56	44	33	33	44	33	33	22	67	44
		post	0	0	0	0	11	0	0	0	0	0	0	0	0	11	33
	HNSO	pre	67	67	75	75	42	25	50	0	75	75	25	42	92	67	58
		post	8	0	0	0	42	0	0	0	0	0	0	0	17	17	0
	HNSC	pre	60	33	53	100	27	47	53	47	40	20	0	80	40	73	40
		post	0	0	0	20	27	0	0	0	0	0	0	20	20	20	33
	HNSX	pre	100	50	100	100	67	100	100	67	83	100	100	100	67	100	100
		post	50	0	0	17	67	50	0	0	0	0	0	33	33	0	100
	HNTE	pre	100	0	0	0	67	100	67	0	0	0	100	0	0	67	100
		post	33	0	0	0	67	100	0	0	0	0	33	0	0	33	0
	HNOM	pre	100	100	100	100	67	100	100	100	100	100	100	67	100	100	67
		post	67	0	0	0	67	0	0	0	33	0	33	0	67	33	67
	HNDR	pre	100	0	0	67	100	33	67	0	0	0	0	0	67	0	33
		post	67	0	0	33	100	0	0	0	0	0	0	0	33	33	33
	HNSS	pre	33	0	0	0	33	0	67	0	0	0	0	0	100	0	0
		post	33	0	0	0	33	0	0	0	0	0	0	0	33	0	0
	HNCO	pre	0	0	0	0	67	0	0	0	0	0	0	0	0	0	0
		post	0	0	0	33	67	0	0	0	33	0	0	0	0	33	0
	HNFI	pre	100	67	33	100	67	100	100	67	67	67	100	100	0	0	67
		post	33	0	0	67	67	0	0	0	67	0	0	0	33	0	33
	HNPK	pre	100	100	100	100	100	100	100	100	100	100	100	100	100	100	100
		post	100	0	0	0	100	0	0	0	0	0	0	0	100	100	100
	HNNU	pre	100	0	0	0	0	0	100	0	0	0	0	0	0	0	0
		post	100	0	0	0	0	0	100	0	0	0	0	0	0	0	0
	HNFE	pre	0	0	0	0	0	0	0	0	0	0	100	0	0	0	0
		post	0	0	0	0	0	0	0	0	0	0	0	0	0	0	0
	HNWL	pre	0	0	0	100	0	100	0	0	100	100	100	0	0	100	0
		post	0	0	0	100	0	0	100	0	0	0	0	100	0	100	0
	HNWG	pre	100	0	0	0	0	0	0	0	0	0	0	100	0	0	0
		post	0	0	0	0	0	100	0	0	0	100	0	0	0	0	0

pre = pre infusion during an acute episode, post = two weeks post infusion. PF2 = Physical functioning, RF2 = Role Functioning, EF = Emotional Functioning, CF = Cognitive Functioning, SF = Social Functioning, QL2 = Quality of Life, FA = Fatigue, NV = Nausea and Vomiting, PA = Pain, DY = Dyspnoea, SL = Insomnia, AP = Appetite Loss, CO = Constipation, DI= Diarrhoea, FI = Financial difficulties. HNPA = Pain, HNSW = Swallowing, HNSE = Senses Problems, HNSP = Speech Problems, HNSO = Trouble with Social Eating, HNSC = Trouble with Social Contact, HNSX = Less Sexuality, HNTE = Teeth, HNOM = Opening mouth, HNDR = Dry mouth, HNSS = Sticky saliva, HNCO = Coughing, HNFI = Felt ill, HNPK = Pain killers, HNNU = Nutritional supplements, HNFE = Feeding tube, HNWL = Weight loss, HNWG = Weight gain. For OHIP-G14, 0 is the best score achievable, 56 the worst. For QLQ-C30 and QLQ-H&N35, scores can range from 0 to 100.

**Table 2:** Mean scores for Ohip-G14, QLQ-C30 and QLQ-H&N35 (N=15).

Mean scores pre and post infusion				
Questionnaire	scale	time	mean	p-value (2-sided)
OHIP-G14	-	pre	32.66	< .001*
		post	6.73	
QLQ-C30	PF2	pre	65.77	< .001
		post	94.22	
	RF2	pre	20.00	< .001
		post	84.44	
	EF	pre	21.67	< .001
		post	73.89	
	CF	pre	22.22	.002
		post	80.00	
	SF	pre	31.11	< .001
		post	92.22	
	QL2	pre	17.78	< .001
		post	76.11	
	FA	pre	71.11	.003
		post	20.00	
	NV	pre	8.89	1.000
		post	8.89	
	PA	pre	100.00	< .001
		post	24.44	
	DY	pre	8.89	.197
		post	2.22	
	SL	pre	84.44	< .001
		post	24.44	
	AP	pre	53.33	.002
		post	2.22	
	CO	pre	13.33	.285
		post	19.05	
	DI	pre	24.44	.223
		post	13.33	
	FI	pre	22.22	.109
		post	8.89	
QLQ-H&N35	HNPA	pre	43.89	< .001
		post	15.56	
	HNSW	pre	28.33	.005
		post	5.56	
	HNSE	pre	13.33	.083
		post	3.33	
	HNSP	pre	41.48	< .001
		post	3.70	
	HNSO	pre	55.56	< .001
		post	5.56	
	HNSC	pre	47.56	< .001
		post	9.33	
	HNSX	pre	88.89	< .001
		post	23.33	
	HNTE	pre	40.00	< .046
		post	17.78	
	HNOM	pre	93.33	< .001
		post	24.44	
	HNDR	pre	31.11	.176
		post	20.00	
	HNSS	pre	15.56	.461
		post	6.67	
	HNCO	pre	4.44	.059
		post	11.11	
	HNFI	pre	68.89	.005
		post	20.00	
	HNPK	pre	100.00	.002
		post	33.33	
	HNNU	pre	13.33	1.000
		post	13.33	
	HNFE	pre	6.67	.317
		post	0.00	
	HNWL	pre	40.00	.414
		post	26.67	
	HNWG	pre	13.33	1.000
		post	13.33	

pre = pre infusion during an acute episode, post = two weeks post infusion. PF2 = Physical functioning, RF2 = Role Functioning, EF = Emotional Functioning, CF = Cognitive Functioning, SF = Social Functioning, QL2 = Quality of Life, FA = Fatigue, NV = Nausea and Vomiting, PA = Pain, DY = Dyspnoea, SL = Insomnia, AP = Appetite Loss, CO = Constipation, DI= Diarrhoea, FI = Financial difficulties. HNPA = Pain, HNSW = Swallowing, HNSE = Senses Problems, HNSP = Speech Problems, HNSO = Trouble with Social Eating, HNSC = Trouble with Social Contact, HNSX = Less Sexuality, HNTE = Teeth, HNOM = Opening mouth, HNDR = Dry mouth, HNSS = Sticky saliva, HNCO = Coughing, HNFI = Felt ill, HNPK = Pain killers, HNNU = Nutritional supplements, HNFE = Feeding tube, HNWL = Weight loss, HNWG = Weight gain. For OHIP-G14, 0 is the best score achievable, 56 the worst. For QLQ-C30 and QLQ-H&N35, scores can range from 0 to 100. p-values obtained with Wilcoxon test. *p-value obtained with paired sample T-test.
